# Characterization of the Volatile Constituents of Plai (*Zingiber purpureum*) by Gas Chromatography–Mass Spectrometry

**DOI:** 10.3390/molecules29061216

**Published:** 2024-03-08

**Authors:** Yuto Nishidono, Azis Saifudin, Ken Tanaka

**Affiliations:** 1 College of Pharmaceutical Sciences, Ritsumeikan University, Kusatsu 525-8577, Shiga, Japan; ktanaka@fc.ritsumei.ac.jp; 2 Research Organization of Science and Technology, Ritsumeikan University, Kusatsu 525-8577, Shiga, Japan; 3 Faculty of Pharmacy, Universitas Muhammadiyah Surakarta, Sukoharjo 57102, Jawa Tengah, Indonesia; azis.saifudin@ums.ac.id

**Keywords:** *Zingiber purpureum* Roscoe, plai, cassumunar ginger, phenylbutenoids, GC–MS

## Abstract

*Zingiber purpureum* Roscoe, known as plai in Thailand, is a perennial plant of the *Zingiberaceae* family and has traditionally been used in Southeast Asian countries to treat inflammation, pain, and asthma. In this study, we performed the characterization of the volatile constituents in ethyl acetate extracts of plai. Ethyl acetate extracts derived from the rhizomes of plai were subjected to gas chromatography–mass spectrometry, and the key peaks in the total ion current chromatograms were annotated or identified. In total, twenty-one compounds were identified using isolation procedures or standards, and nine compounds were annotated by comparing their Kovats retention index (RI) and electron ionization (EI) mass spectra with those in the literature. Most of the identifications were inconsistent with the tentative annotations found via library search and suggested that some peaks were incorrectly assigned in previous studies. Thus, to avoid further misannotations and contribute to the research on dereplication, the RI value, EI mass spectral data, and NMR spectroscopy data of the isolated compounds are reported.

## 1. Introduction

The goal of metabolomics research on medicinal plants is to comprehensively and accurately identify all low-molecular-weight metabolites [1], thus providing an effective approach to evaluating the quality of medicinal plants [2]. These studies are mainly based on targeted and untargeted analyses [1]. In untargeted analyses, annotation and identification are critical to converting metabolomics data into meaningful biological knowledge [3]. However, especially in studies on medicinal plants, the process of annotation and identification of metabolites remains a major bottleneck due to the limited data in libraries and lack of standards [1,4].

In 2007, four different levels of metabolite identification were defined by the chemical analysis working group of the Metabolomics Standards Initiative (MSI) [5,6], namely, the identified compounds (level 1), putatively annotated compounds (level 2), putatively characterized compound classes (level 3), and unknown compounds (level 4). Recently, level 0, which includes compounds identified via isolation and full stereochemical characterization, was established as a new confidence level of metabolite identification [6,7]. In many studies, authentic standards were not used; therefore, annotations (levels 2 and 3) and not identifications (levels 0 and 1) were achieved [8].

*Zingiber purpureum* Roscoe (syn *Z. cassumunar* Roxb.) is a perennial plant in the *Zingiberaceae* known as plai (phlai) in Thailand and bangle or bengle in Indonesia [9,10,11]. Over the past two decades, *Z. montanum* (J.Koenig) Link ex A.Dietr. has been accepted as the scientific name for plai (cassumunar ginger), whereas Bai et al. recently proposed that *Z. purpureum* Roscoe is the correct name for this plant [12]. This herb is widely used as a remedy or component of herbal recipes in Asian countries [9]. In Thailand, it is used as the main component in massage oil to relieve muscle pain and is consumed to relieve asthma [9,13]. In the Thai Herbal Pharmacopoeia, plai is listed as an anti-inflammatory, counter-irritant, and mosquito-repellent herb [14]. In fact, products using plai oil are currently made and distributed to alleviate muscle pain [15]. In Indonesia, bangle has been used to relieve colic in children [13], to treat abdominal obesity in postpartum women [16], and as a vermifuge and an analeptic for the uterus [17]. Scientific studies have revealed the bioactivities of the extracts or fractions of this plant behind these traditional uses, such as antioxidant, anti-inflammatory, antifungal, antimicrobial, anti-asthma, neuroprotective, anticancer, antiaging, and skin whitening effects [18].

Plai contains several types of secondary metabolites. Among them, phenylbutenoids, curcuminoids, and essential oil constituents are the major bioactive compounds [18]. In particular, phenylbutenoids are major characteristic compounds of this herb and show various biological activities, including anti-asthma, anticancer, anti-inflammatory, chondroprotective, and melanogenic effects [18,19]. Owing to their volatility, gas chromatography–mass spectrometry (GC–MS) is often employed for the analysis of the extracts and oils of plai [15,20,21,22,23,24,25,26,27,28,29,30,31,32,33,34,35,36,37,38,39]. Although these studies contributed to the elucidation of chemical constituents in plai, some peaks are still unidentified, and some discrepancies in peak annotations are found in the literature.

In this study, to confirm or revise the peak annotation of the volatile constituents in plai extracts, we performed their characterization. In particular, the compounds corresponding to key peaks were isolated, and their structures were elucidated based on NMR spectroscopic data. Finally, Kovats retention index (RI) value, electron ionization (EI) mass spectral data, and NMR data of the isolated compounds are reported to contribute to further studies on the chemical constituents of plai.

## 2. Results and Discussion

**Figure 1:**
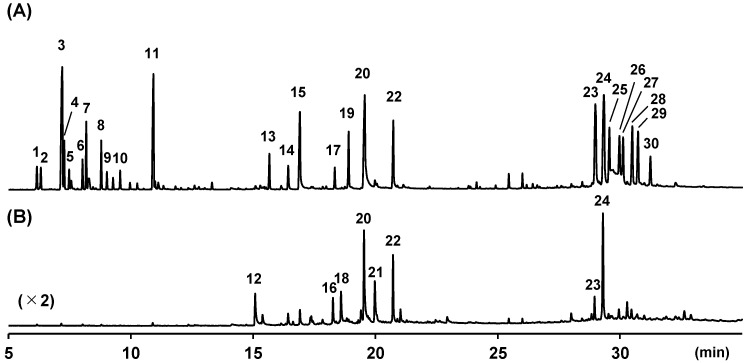
Gas chromatography–mass spectrometry total ion current chromatograms of ethyl acetate extracts of plai purchased in Thailand (**A**) and Indonesia (**B**). The numbers in the figure refer to the structures in Figure 2.

**Figure 2:**
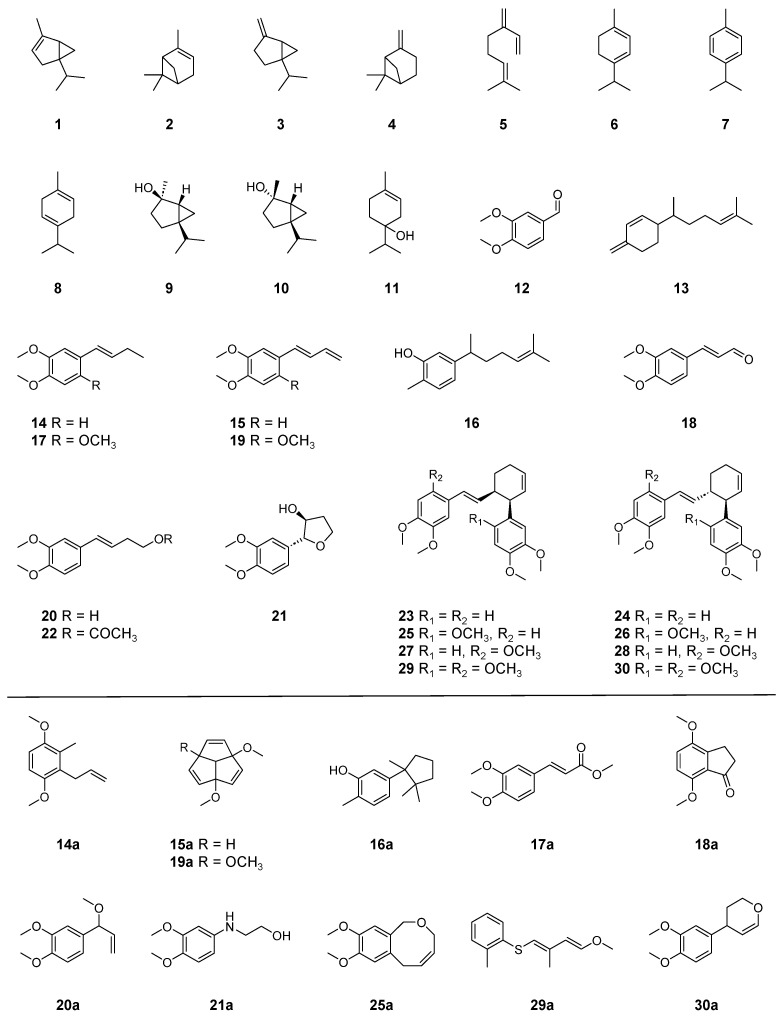
Chemical structures of annotated or identified compounds.

Previous studies have shown that essential oils obtained through the hydrodistillation of plai contained phenylbutenoid monomers [26], and the ethyl acetate fraction of the 70% ethanol extract of plai contained phenylbutenoid monomers and dimers [33]. Therefore, herein, the dried rhizomes of two plai samples purchased in Thailand and Indonesia were extracted using ethyl acetate, and then the extracts were subjected to GC–MS (Figure 1). Thirty major peaks were detected in the GC–MS total ion current (TIC) chromatograms of the extracts, and their tentative annotation was performed via library search using the Wiley 9 database (Table 1 and Figure 2).

Next, verification of the tentative annotations was conducted. The annotation of peaks 1, 2, 4–8, and 13 was verified by comparing the EI mass spectrum and RI value with those reported [40]. Peaks 3 and 11 were identified as sabinene (**3**) and terpinen-4-ol (**11**), respectively, using standards. In addition, peak 12 was assigned to 3,4-dimethoxybenzaldehyde (**12**) via isolation [41]. Therefore, the annotation and identification of these peaks was consistent with the annotation found via library search.

Peaks 9 and 10 were tentatively annotated as 4-thujanol, which is also known as sabinene hydrate. The configuration of the purchased standard was confirmed as a *trans*-configuration by comparing its NMR spectroscopic data with those of *trans*- and *cis*-4-thujanol [42,43]. According to the RI value and EI mass spectral data of the standard, peak 9 was finally identified as *trans*-4-thujanol (**9**). Moreover, additional RI values revealed that peak 10 was *cis*-4-thujanol (**10**) [40]. These results confirm that some peaks were previously misannotated [20,21,22,23,24,25]. In particular, *trans*-4-thujanol (*trans*-sabinene hydrate) and *cis*-4-thujanol (*cis*-sabinene hydrate) were reversely annotated to their corresponding peaks, most likely because the compound recently known as *trans*-sabinene hydrate was classically named as *cis*-sabinene hydrate [44]. Following the suggestion by Mladenović and Radulović [45], *trans*-4-thujanol (**9**) and *cis*-4-thujanol (**10**) in this study are also shown as (1S*,3R*,4R*)-4-thujanol (**9**) and (1S*,3R*,4S*)-4-thujanol (**10**), respectively.

Peaks 14 and 17 were tentatively annotated as 1,4-dimethoxy-2-methyl-3-(2-propen-1-yl)benzene (**14a**) and methyl 3,4-dimethoxycinnamate (**17a**), respectively, via a library search. However, their EI mass spectrum and RI value were similar to those of phenylbutenoids [26]. To investigate these differences, the compounds corresponding to peaks 14 and 17 were isolated, and their structures were established on the basis of the NMR data [46]. Therefore, peaks 14 and 17 were finally assigned to (*E*)-1-(3′,4′-dimethoxyphenyl)but-1-ene (**14**) and (*E*)-1-(2′,4′,5′-trimethoxyphenyl)but-1-ene (**17**), respectively.

**Figure 3:**
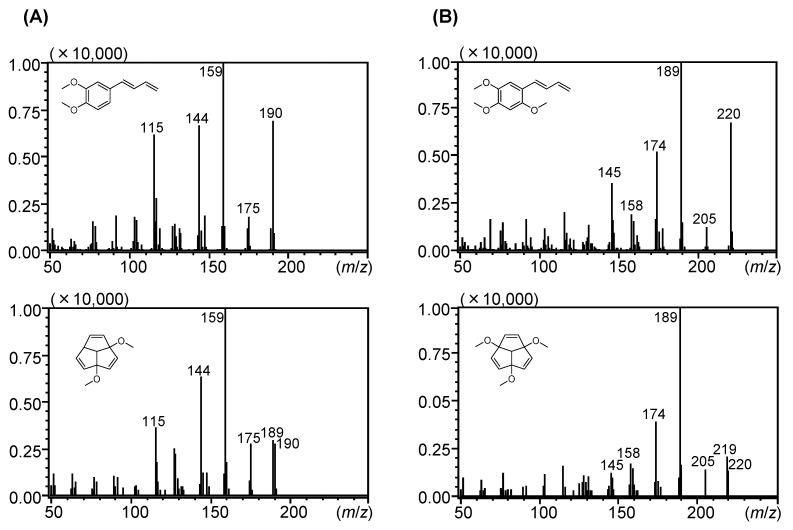
Comparison between the EI mass spectra of (**A**) (*E*)-1-(3′,4′-dimethoxyphenyl)buta-1,3-diene (**15**) and 1,4-dimethoxytriquinacene (**15a**) and (**B**) (*E*)-1-(2′,4′,5′-trimethoxyphenyl)buta-1,3-diene (**19**) and 1,4,7-trimethoxytriquinacene (**19a**). EI-MS mass spectra of **15a** and **19a** were obtained from the Wiley 9 database.

The EI mass spectrum of peak 15 showed the molecular ion peak at 190 (*m*/*z*), and peak 15 was tentatively annotated as 1,4-dimethoxytriquinacene (**15a**) via a library search. Previous studies also reported the presence of a compound annotated as 1,4-dimethoxytriquinacene (**15a**) [25,27,28,29,30]. In addition, Mektrirat et al. annotated the compound affording the molecular ion peak at 190 (*m*/*z*), which eluted after β-sesquiphellandrene, as 1,2-dimethyl-6-nitroindolizine on the basis of the mass spectral libraries [31]. However, the EI mass spectrum of peak 15 was consistent with that of (*E*)-1-(3′,4′-dimethoxyphenyl)buta-1,3-diene (**15**) [26]. Therefore, the compound corresponding to peak 15 was isolated to clarify its structure, finding that peak 15 indeed corresponds to (*E*)-1-(3′,4′-dimethoxyphenyl)buta-1,3-diene (**15**) [47]. Similarly, peak 19 was tentatively annotated as 1,4,7-trimethoxytriquinacene (**19a**). Although this annotation was consistent with previous studies [25,27,28,29], the present study shows that the compound corresponding to peak 19 was (*E*)-1-(2′,4′,5′-trimethoxyphenyl)buta-1,3-diene (**19**) [48]. To avoid further misannotation, the differences in the EI mass spectrum between phenylbutadienes (identified compounds) and triquinacenes (misassigned compounds) were clarified. As shown in Figure 3, phenylbutadienes and triquinacenes can be discriminated on the basis of the ratio of their molecular ion peaks [M]^+^ and their deprotonated molecular ion peaks [M − H]^+^.

The EI mass spectrum of peak 16 showed the molecular ion peak at 218 (*m*/*z*) and the base ion peak at 136 (*m*/*z*). It was annotated as δ-cuparenol (**16a**) via a library search with 80% similarity, and Risnawati et al. could not identify this peak [32]. In this study, a search in the Adams database revealed that xanthorrhizol showed these features [40], which is consistent with other studies [49,50]. Accordingly, using the standard, peak 16 was finally identified as xanthorrhizol (**16**), which is reported as a component of *Z. purpureum* Roscoe for the first time.

Peak 18 was tentatively annotated as 4,7-dimethoxy-1-indanone (**18a**) and was finally identified as (*E*)-3-(3′,4′-dimethoxyphenyl)propenal (**18**) [51].

Peaks 20 and 22 were finally identified as (*E*)-4-(3′,4′-dimethoxyphenyl)but-3-en-1-ol (**20**) and (*E*)-4-(3′,4′-dimethoxyphenyl)but-3-en-1-yl acetate (**22**), respectively [52]. These identifications were consistent with previous studies [25,32,33].

In the EI mass spectrum of peak 21, the molecular ion peak was detected at 224 (*m*/*z*). To date, some compounds consistent with this result have been isolated from plai, including (*E*)-4-(3,4-dimethoxyphenyl)but-3-ene-1,2-diol [52] and cassumunols G and H [53]. Among them, only the EI mass spectrum of (*E*)-4-(3,4-dimethoxyphenyl)but-3-ene-1,2-diol was reported [52], although it was not consistent with that of peak 21. Thus, cassumunol H (**21**) was isolated and assigned to peak 21 [53].

**Figure 4:**
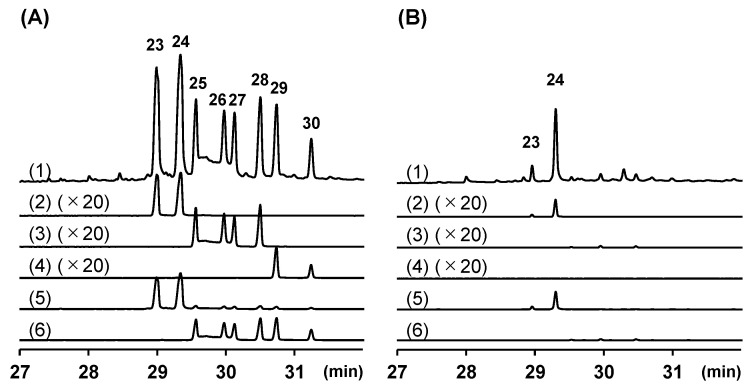
Enlarged gas chromatography–mass spectrometry total ion current (TIC) and extracted ion current chromatograms of ethyl acetate extracts of plai purchased in Thailand (**A**) and Indonesia (**B**). (1) TIC, (2) *m*/*z* 380 ([M]^+^ of compounds **23** and **24**), (3) *m*/*z* 410 ([M]^+^ of compounds **25**–**28**), (4) *m*/*z* 440 ([M]^+^ of compounds **29** and **30**), (5) *m*/*z* 190, (6) *m*/*z* 220. The numbers in the figure refer to the structures in Figure 2.

Peaks 23 and 24 were tentatively annotated as the phenylbutenoid monomer (*E*)-1-(3′,4′-dimethoxyphenyl)buta-1,3-diene via a library search. The EI mass spectra of these peaks were almost the same, and the molecular ion and base ion peaks were detected at 380 (*m*/*z*) and 190 (*m*/*z*), respectively (Figure 4 and Figure S1). Risnawati et al. suggested that their corresponding compounds are *cis*-l,2-bis[(*E*)-3,4-dimethoxystyryl]cyclobutene, *cis*-banglene (*cis*-3-(3′,4′-dimethoxyphenyl)-4-[(*E*)-3‴,4‴-dimethoxystyryl]cyclohex-1-ene), or *trans*-banglene (*trans*-3-(3′,4′-dimethoxyphenyl)-4-[(*E*)-3‴,4‴-dimethoxystyryl]cyclohex-1-ene) [32]. In this study, the compounds corresponding to peaks 23 and 24 were isolated, and their structures were elucidated via NMR analysis [46], allowing the assignment of these peaks to *cis*-banglene (**23**) and *trans*-banglene (**24**), respectively. Similarly, the EI mass spectra of peaks 29 and 30 showed the molecular ion peak at 440 (*m*/*z*) and the base ion peak at 220 (*m*/*z*) (Figure 4 and Figure S1). These peaks were finally assigned to 2′,2‴-dimethoxy *cis*-banglene (*cis*-3-(2′,4′,5′-trimethoxyphenyl)-4-[(*E*)-2‴,4‴,5‴-trimethoxystyryl]cyclohex-1-ene) (**29**) and 2′,2‴-dimethoxy *trans*-banglene (*trans*-3-(2′,4′,5′-trimethoxyphenyl)-4-[(*E*)-2‴,4‴,5‴-trimethoxystyryl]cyclohex-1-ene) (**30**), respectively [46,54].

Peaks 25–28 showed almost the same EI mass spectra, in which the molecular ion peak and the base ion peak were detected at 410 (*m*/*z*) and 220 (*m*/*z*), respectively (Figure 4 and Figure S1), indicating that these peaks correspond to banglenes with an additional methoxy group. Because the EI mass spectra could not reveal the position of methoxy groups, the corresponding compounds were isolated, and their structures were elucidated [46,55]. Accordingly, peaks 25–28 were finally identified, as shown in Table 1 and Figure 2.

Plai samples purchased in Thailand and Indonesia showed different volatile compositions (Figure 1). The relative amounts of compounds **1**–**30** in ethyl acetate extracts calculated according to the GC–MS peak areas are listed in Table S1. 3,4-Dimethoxybenzaldehyde (**12**) and (*E*)-3-(3′,4′-dimethoxyphenyl)propenal (**18**) were only detected in the ethyl acetate extract obtained from the rhizomes of the sample purchased in Indonesia. In addition, the extract of the same sample showed a low intensity of (*E*)-1-(3′,4′-dimethoxyphenyl)buta-1,3-diene (**15**). These observations were consistent with Seaho’s suggestion that these aldehydes **12** and **18** are produced via the oxidative double-bond cleavage of (*E*)-1-(3′,4′-dimethoxyphenyl)buta-1,3-diene (**15**) during storage [19]. Therefore, different volatile compositions between the two analyzed samples may have resulted from the differences in storage methods and duration, in addition to geographical variations.

In total, twenty-one compounds, including fifteen phenylbutenoids and one phenylbutanoid, were identified, and nine compounds were annotated. The results indicate that some peaks were incorrectly assigned in previous investigations [20,21,22,23,24,25,27,28,29,30,31,34]. To avoid further misannotations and to contribute to the research on dereplication, the RI value, EI mass spectral data, and NMR data of the isolated compounds are summarized in Table 1 and in the Materials and Methods section. Some of these data, such as the RI value of compounds **23**–**30** and the EI mass spectral data of compound **21**, are reported for the first time.

## 3. Materials and Methods

### 3.1. General Experimental Procedures

NMR spectra were recorded using a JNM-ECZ500R spectrometer (JEOL Ltd., Tokyo, Japan). Tetramethylsilane (0.00 ppm for ^1^H NMR) and the solvent peak (77.0 ppm for ^13^C NMR) in chloroform-*d* were used as internal standards. GC–MS was performed on a Shimadzu GCMS-QP2010 (Shimadzu Corporation, Kyoto, Japan). Medium-pressure liquid chromatography (MPLC) was performed using a Yamazen pump 540 (Yamazen Corporation, Osaka, Japan) equipped with a universal column premium silica gel or a universal column ODS (Yamazen Corporation). Semipreparative high-performance liquid chromatography (HPLC) was performed on a Shimadzu prominence HPLC system equipped with a CBM-20A communication bus module, a LC-20AR liquid chromatograph, a SIL-10AF auto sampler, an SPD-20A UV/Vis detector, and an FRC-10A fraction collector (Shimadzu Corporation). The flow rate and wavelength were set at 5 mL/min and 254 nm, respectively. Separation was performed using a Cosmosil 5C_18_ MS-II packed column (5 μm, 10 mm × 250 mm, Nacalai Tesque, Inc., Kyoto, Japan) or a Cosmosil Cholester column (5 μm, 10 mm × 250 mm, Nacalai Tesque, Inc.). Thin-layer chromatography was conducted on precoated silica gel 60 F_254_ or RP-18 F_254_ plates (Merck, Darmstadt, Germany).

### 3.2. Plant Materials

Dried rhizomes of *Zingiber purpureum* Roscoe (*Zingiberaceae*) were purchased from the crude drug market in Surakarta, Indonesia, in August 2017 and Bangkok, Thailand, in July 2018. These crude drugs were authenticated by one of the authors (A.S.). A voucher specimen (RIN-170102 and 180107) has been deposited at the Museum of Materia Medica, Ritsumeikan University (Kusatsu, Shiga, Japan).

### 3.3. Chemicals

A standard alkane mixture (C9–C40) was purchased from GL Sciences (Tokyo, Japan). Sabinene (**3**), *trans*-4-thujanol (**9**), terpinen-4-ol (**11**), and xanthorrhizol (**16**) were purchased from Nacalai Tesque Inc. Other analytical-grade chemicals and chromatographic solvents were purchased from Nacalai Tesque Inc. or FUJIFILM Wako Pure Chemical Corporation (Osaka, Japan).

### 3.4. Extraction and Isolation

The dried rhizomes of plai (111 g) purchased in Thailand were pulverized and extracted with ethyl acetate under reflux (3 × 500 mL, each 1 h). After the solvent was evaporated in vacuo, 11.0 g of crude extract was obtained. A portion of the ethyl acetate extract (9.58 g) was chromatographed on silica gel using an *n*-hexane/ethyl acetate solvent system (70:30–0:100) to give 15 fractions. Fr. 2 (526.4 mg) and Fr. 4 (155.8 mg) were subjected to MPLC with chloroform and semipreparative HPLC (Cosmosil Cholester column) with an isocratic mobile phase of 80% aq. acetonitrile, yielding compounds **14** (30.1 mg, *t*_R_ = 4.7 min) and **17** (33.6 mg, *t*_R_ = 4.7 min), respectively. Fr. 3 (555.5 mg) and 5 (279.1 mg) were purified via MPLC (90% MeOH) to yield compounds **15** (312.8 mg) and **19** (106.8 mg), respectively. An aliquot of Fr. 7 (112.6 mg) was further purified via semipreparative HPLC (Cosmosil Cholester column) eluted with 80% acetonitrile to afford compounds **24** (50.4 mg, *t*_R_ = 7.3 min) and **23** (46.1 mg, *t*_R_ = 8.1 min). The same procedure was performed for the purification of Fr. 10 (121.8 mg), giving compounds **30** (16.4 mg, *t*_R_ = 7.1 min) and **29** (49.9 mg, *t*_R_ = 7.6 min). Fr. 8 (105.7 mg) was separated by semipreparative HPLC (5C_18_ MS-II packed column, acetonitrile–water, 8:2) to yield compounds **26** (29.8 mg, *t*_R_ = 7.6 min) and **25** (69.4 mg, *t*_R_ = 8.3 min). Fr. 9 (103.8 mg) was also purified by semipreparative HPLC (5C_18_ MS-II packed column, acetonitrile–water, 7:3) to yield compounds **28** (51.5 mg, *t*_R_ = 9.0 min) and **27** (34.8 mg, *t*_R_ = 10.1 min).

Similarly, the ethyl acetate extract (8.74 g) was obtained from dried rhizomes of plai (105 g) purchased in Indonesia. A portion of the extract (1.30 g) was subjected to MPLC with an ODS column eluted by 80% methanol to give six fractions (Fr. 1–Fr. 6). Fr. 1 (26.6 mg) was separated by preparative TLC (chloroform:methanol = 95:5) to afford compound **21** (1.8 mg). Fr. 2 (245.0 mg) was separated using a universal column premium silica gel with an elution of *n*-hexane–ethyl acetate (6:4 *v*/*v*) to give compounds **12** (15.3 mg), **18** (11.8 mg), and **20** (40.8 mg). Fr. 3 (118.2 mg) was purified under the same conditions, yielding compound **22** (17.5 mg).

#### 3.4.1. 3,4-Dimethoxybenzaldehyde (**12**)

^1^H NMR (500 MHz, CDCl_3_): δ_H_ 3.95 (3H, s), 3.98 (3H, s), 6.99 (1H, d, *J* = 8.6 Hz), 7.42 (1H, d, *J* = 1.7 Hz), 7.47 (1H, dd, *J* = 8.6, 1.7 Hz), 9.86 (1H, s); ^13^C NMR (125 MHz, CDCl_3_): δ_C_ 56.0, 56.1, 108.8, 110.3, 126.9, 130.1, 149.5, 154.4, 190.9; EI-MS: *m*/*z* 166 (100), 165 (65), 151 (14), 95 (50), 80 (10), 79 (26), 77 (43), 67 (13), 65 (19), 63 (12), 52 (13), 51 (25).

#### 3.4.2. (*E*)-1-(3′,4′-Dimethoxyphenyl)but-1-ene (**14**)

^1^H NMR (500 MHz, CDCl_3_): δ_H_ 1.09 (3H, t, *J* = 7.5 Hz), 2.22 (2H, m), 3.87 (3H, s), 3.90 (3H, s), 6.14 (1H, dt, *J* = 15.8, 6.5 Hz), 6.32 (1H, dt, *J* = 15.8, 1.5 Hz), 6.80 (1H, d, *J* = 8.2 Hz), 6.87 (1H, dd, *J* = 8.2, 2.0 Hz), 6.92 (1H, d, *J* = 2.0 Hz); ^13^C NMR (125 MHz, CDCl_3_): δ_C_ 13.8, 26.0, 55.7, 55.9, 108.3, 111.1, 118.7, 128.3, 130.8, 131.1, 148.1, 148.9; EI-MS: *m*/*z* 192 (99), 178 (12), 177 (100), 162 (16), 161 (72), 149 (41), 147 (16), 146 (56), 145 (19), 133 (11), 131 (28), 121 (27), 119 (23), 117 (47), 116 (13), 115 (44), 107 (32), 105 (21), 103 (29), 93 (11), 91 (70), 89 (11), 79 (19), 78 (15), 77 (39), 65 (26), 63 (13), 55 (20), 51 (20).

#### 3.4.3. (*E*)-1-(3′,4′-Dimethoxyphenyl)buta-1,3-diene (**15**)

^1^H NMR (500 MHz, CDCl_3_): δ_H_ 3.89 (3H, s), 3.92 (3H, s), 5.13 (1H, brd, *J* = 10.9 Hz), 5.30 (1H, brd, *J* = 16.3 Hz), 6.49 (1H, ddd, *J* = 16.3, 10.9, 10.6 Hz), 6.51 (1H, d, *J* = 15.0 Hz), 6.67 (1H, dd, *J* = 15.0, 10.6 Hz), 6.82 (1H, d, *J* = 8.4 Hz), 6.95 (1H, dd, *J* = 8.4, 1.7 Hz), 6.96 (1H, d, *J* = 1.7 Hz); ^13^C NMR (125 MHz, CDCl_3_): δ_C_ 55.8, 55.9, 108.5, 111.1, 116.7, 119.8, 127.8, 130.2, 132.6, 137.2, 148.9, 149.0; EI-MS: *m*/*z* 190 (69), 189 (12), 175 (18), 174 (12), 160 (13), 159 (100), 158 (13), 147 (19), 145 (10), 144 (67), 131 (12), 128 (14), 127 (13), 119 (12), 117 (28), 116 (15), 115 (62), 104 (16), 103 (18), 91 (18), 78 (13), 77 (15), 51 (12).

#### 3.4.4. (*E*)-1-(2′,4′,5′-Trimethoxyphenyl)but-1-ene (**17**)

^1^H NMR (500 MHz, CDCl_3_): δ_H_ 1.10 (3H, t, *J* = 7.5 Hz), 2.24 (2H, m), 3.82 (3H, s), 3.87 (3H, s), 3.89 (3H, s), 6.13 (1H, dt, *J* = 15.9, 6.6 Hz), 6.50 (1H, s), 6.64 (1H, dt, *J* = 15.9, 1.5 Hz), 6.97 (1H, s); ^13^C NMR (125 MHz, CDCl_3_): δ_C_ 13.9, 26.4, 56.1, 56.4, 56.7, 97.8, 109.5, 118.8, 122.7, 131.3, 143.3, 148.7, 150.7; EI-MS: *m*/*z* 222 (100), 207 (66), 191 (41), 179 (32), 177 (16), 176 (54), 175 (14), 161 (24), 151 (23), 149 (15), 147 (25), 137 (21), 133 (12), 132 (10), 121 (14), 117 (11), 115 (15), 107 (13), 105 (12), 103 (12), 91 (27), 79 (13), 77 (23), 69 (18), 55 (12).

#### 3.4.5. (*E*)-3-(3′,4′-Dimethoxyphenyl)propenal (**18**)

^1^H NMR (500 MHz, CDCl_3_): δ_H_ 3.93 (3H, s), 3.94 (3H, s), 6.62 (1H, dd, *J* = 15.8, 7.7 Hz), 6.91 (1H, d, *J* = 8.3 Hz), 7.08 (1H, d, *J* = 2.0 Hz), 7.17 (1H, dd, *J* = 8.3, 2.0 Hz), 7.43 (1H, d, *J* = 15.8 Hz), 9.67 (1H, d, *J* = 7.7 Hz); ^13^C NMR (125 MHz, CDCl_3_): δ_C_ 55.9, 56.0, 109.7, 111.0, 123.4, 126.6, 127.0, 149.3, 151.9, 152.9, 193.6; EI-MS: *m*/*z* 192 (62), 191 (13), 177 (24), 162 (13), 161 (100), 149 (25), 138 (11), 133 (19), 121 (29), 118 (14), 106 (14), 105 (19), 103 (23), 93 (12), 91 (48), 89 (18), 79 (13), 78 (27), 77 (53), 65 (16), 63 (14), 52 (11), 51 (25).

#### 3.4.6. (*E*)-1-(2′,4′,5′-Trimethoxyphenyl)buta-1,3-diene (**19**)

^1^H NMR (500 MHz, CDCl_3_): δ_H_ 3.84 (3H, s), 3.88 (3H, s), 3.90 (3H, s), 5.10 (1H, brd, *J* = 10.2 Hz), 5.28 (1H, brd, *J* = 16.8 Hz), 6.50 (1H, s), 6.53 (1H, ddd, *J* = 16.8, 10.5, 10.2 Hz), 6.68 (1H, dd, *J* = 15.8, 10.5 Hz), 6.86 (1H, d, *J* = 15.8 Hz), 7.01 (1H, s); ^13^C NMR (125 MHz, CDCl_3_): δ_C_ 56.1, 56.4, 56.7, 97.6, 109.3, 116.0, 117.9, 127.1, 128.0, 138.0, 143.3, 149.6, 151.6; EI-MS: *m*/*z* 220 (67), 205 (12), 190 (15), 189 (100), 177 (12), 174 (52), 173 (17), 159 (15), 158 (19), 146 (16), 145 (36), 131 (14), 115 (20), 103 (12), 91 (17), 77 (15), 75 (11), 69 (17).

#### 3.4.7. (*E*)-4-(3′,4′-Dimethoxyphenyl)but-3-en-1-ol (**20**)

^1^H NMR (500 MHz, CDCl_3_): δ_H_ 2.47 (2H, dtd, *J* = 7.2, 6.2, 1.2 Hz), 3.76 (2H, t, *J* = 6.2 Hz), 3.88 (3H, s), 3.90 (3H, s), 6.08 (1H, dt, *J* = 15.9, 7.2 Hz), 6.44 (1H, dt, *J* = 15.9, 1.2 Hz), 6.81 (1H, d, *J* = 8.2 Hz), 6.89 (1H, dd, *J* = 8.2, 1.9 Hz), 6.93 (1H, d, *J* = 1.9 Hz); ^13^C NMR (125 MHz, CDCl_3_): δ_C_ 36.3, 55.8, 55.9, 62.1, 108.4, 111.0, 119.1, 124.3, 130.3, 132.5, 148.5, 148.9; EI-MS: *m*/*z* 208 (39), 178 (12), 177 (100), 147 (12), 146 (48), 131 (16), 91 (10).

#### 3.4.8. Cassumunol H (**21**)

^1^H NMR (500 MHz, CDCl_3_): δ_H_ 1.96 (1H, dddd, *J* = 13.0, 7.3, 4.0, 3.7 Hz), 2.22 (1H, dddd, *J* = 13.0, 8.6, 8.6, 6.3 Hz), 3.88 (3H, s), 3.89 (3H, s), 4.13 (1H, ddd, *J* = 8.6, 8.6, 7.3 Hz), 4.21 (1H, ddd, *J* = 8.6, 8.6, 4.0 Hz), 4.28 (1H, ddd, *J* = 6.3, 3.7, 3.6 Hz), 4.71 (1H, d, *J* = 3.6 Hz), 6.85 (1H, d, *J* = 8.0 Hz), 6.89 (1H, brs), 6.89 (1H, m); ^13^C NMR (125 MHz, CDCl_3_): δ_C_ 34.2, 55.9, 55.9, 67.0, 78.8, 87.2, 108.6, 111.1, 117.7, 133.1, 148.4, 149.0; EI-MS: *m*/*z* 224 (47), 167 (97), 166 (46), 165 (36), 152 (11), 151 (35), 139 (100), 124 (27), 109 (12), 108 (18), 107 (14), 95 (17), 91 (10), 79 (14), 77 (26), 65 (13), 57 (24).

#### 3.4.9. (*E*)-4-(3′,4′-Dimethoxyphenyl)but-3-en-1-yl acetate (**22**)

^1^H NMR (500 MHz, CDCl_3_): δ_H_ 2.07 (3H, s), 2.53 (2H, dtd, *J* = 7.0, 6.8, 1.4 Hz), 3.88 (3H, s), 3.91 (3H, s), 4.18 (2H, t, *J* = 6.8 Hz), 6.04 (1H, dt, *J* = 15.8, 7.0 Hz), 6.41 (1H, dt, *J* = 15.8, 1.4 Hz), 6.81 (1H, d, *J* = 8.2 Hz), 6.89 (1H, dd, *J* = 8.2, 1.9 Hz), 6.91 (1H, d, *J* = 1.9 Hz); ^13^C NMR (125 MHz, CDCl_3_): δ_C_ 21.0, 32.3, 55.8, 55.9, 63.8, 108.5, 111.0, 119.1, 123.6, 130.3, 132.0, 148.5, 148.9, 171.2; EI-MS: *m*/*z* 250 (9), 191 (10), 190 (66), 189 (11), 177 (13), 175 (25), 160 (14), 159 (100), 147 (27), 146 (35), 144 (24), 131 (17), 119 (12), 117 (17), 115 (22), 103 (12), 91 (15).

#### 3.4.10. *cis*-Banglene (**23**)

^1^H NMR (500 MHz, CDCl_3_): δ_H_ 1.63 (2H, m), 2.19 (1H, m), 2.26 (1H, m), 2.71 (1H, m), 3.51 (1H, brs), 3.76 (3H, s), 3.83 (3H, s), 3.86 (3H, s), 3.86 (3H, s), 5.58 (1H, dd, *J* = 15.8, 9.2), 5.80 (1H, m, *J* = 10.0 Hz), 5.98 (1H, m, *J* = 10.0 Hz), 6.26 (1H, d, *J* = 15.8), 6.69 (1H, d, *J* = 1.9), 6.73 (1H, brs), 6.75 (1H, dd, *J* = 8.2, 1.9), 6.76 (2H, m), 6.80 (1H, d, *J* = 8.2); ^13^C NMR (125 MHz, CDCl_3_): δ_C_ 24.2, 24.8, 42.6, 45.7, 55.7, 55.7, 55.8, 55.9, 108.5, 110.2, 111.0, 113.5, 118.7, 121.9, 128.0, 128.4, 129.1, 131.0, 132.4, 133.7, 147.4, 148.0, 148.1, 148.8; EI-MS: *m*/*z* 380 (7), 191 (13), 190 (100), 175 (15), 160 (10), 159 (80), 144 (13), 115 (10).

#### 3.4.11. *trans*-Banglene (**24**)

^1^H NMR (500 MHz, CDCl_3_): δ_H_ 1.67 (1H, m), 1.92 (1H, m), 2.21 (2H, m), 2.35 (1H, m), 3.18 (1H, m, *J* = 8.6), 3.83 (3H, s), 3.85 (3H, s), 3.86 (3H, s), 3.88 (3H, s), 5.68 (1H, m, *J* = 10.0 Hz), 5.90 (1H, m, *J* = 10.0 Hz), 6.02 (1H, dd, *J* = 15.9, 7.3), 6.09 (1H, d, *J* = 15.9), 6.70 (1H, d, *J* = 1.9), 6.73 (1H, dd, *J* = 8.2, 1.9), 6.77 (1H, d, *J* = 8.2), 6.78 (1H, d, *J* = 8.2), 6.81 (1H, dd, *J* = 8.2, 1.9), 6.82 (1H, d, *J* = 1.9); ^13^C NMR (125 MHz, CDCl_3_): δ_C_ 24.5, 27.8, 45.4, 48.0, 55.8, 55.8, 58.8, 55.9, 108.5, 110.7, 111.0, 111.5, 118.7, 120.4, 127.6, 128.8, 130.2, 130.9, 132.1, 137.5, 147.2, 148.2, 148.5, 148.8; EI-MS: *m*/*z* 380 (6), 191 (14), 190 (100), 175 (16), 160 (10), 159 (81), 144 (13), 115 (11).

#### 3.4.12. 2′-Methoxy *cis*-banglene (**25**)

^1^H NMR (500 MHz, CDCl_3_): δ_H_ 1.73 (2H, m), 2.17 (1H, m), 2.27 (1H, m), 2.76 (1H, m), 3.67 (3H, s), 3.79 (3H, s), 3.84 (3H, s), 3.85 (3H, s), 3.86 (3H, s), 4.10 (1H, brs), 5.70 (1H, m, *J* = 10.0 Hz), 5.84 (1H, dd, *J* = 15.9, 8.5), 5.97 (1H, m, *J* = 10.0 Hz), 6.10 (1H, d, *J* = 15.9), 6.45 (1H, s), 6.70 (1H, brs), 6.73 (2H, m), 6.75 (1H, s); ^13^C NMR (125 MHz, CDCl_3_): δ_C_ 24.0, 25.2, 37.1, 41.2, 55.7, 55.9, 56.1, 56.3, 56.7, 96.9, 108.5, 111.1, 114.8, 118.5, 122.0, 128.1, 128.2, 129.6, 131.4, 131.5, 142.2, 147.8, 147.9, 148.7, 151.6; EI-MS: *m*/*z* 410 (9), 221 (15), 220 (100), 205 (10), 190 (16), 189 (76), 174 (12), 159 (14).

#### 3.4.13. 2′-Methoxy *trans*-banglene (**26**)

^1^H NMR (500 MHz, CDCl_3_): δ_H_ 1.69 (1H, m), 1.87 (1H, m), 2.21 (2H, m), 2.36 (1H, m), 3.71 (3H, s), 3.74 (1H, m, *J* = 8.6), 3.84 (3H, s), 3.86 (3H, s), 3.86 (3H, s), 3.88 (3H, s), 5.60 (1H, m, *J* = 10.0 Hz), 5.90 (1H, m, *J* = 10.0 Hz), 6.07 (2H, m), 6.45 (1H, s), 6.73 (1H, s), 6.76 (1H, d, *J* = 8.6), 6.79 (1H, dd, *J* = 8.6, 1.7), 6.83 (1H, d, *J* = 1.7); ^13^C NMR (125 MHz, CDCl_3_): δ_C_ 24.3, 27.9, 39.6, 45.1, 55.7, 55.9, 56.1, 56.6, 56.6, 97.4, 108.5, 111.0, 112.6, 118.7, 124.8, 127.6, 128.0, 130.3, 131.2, 132.6, 142.9, 147.6, 148.0, 148.8, 151.4; EI-MS: *m*/*z* 410 (10), 221 (15), 220 (100), 205 (11), 190 (15), 189 (80), 174 (12), 159 (14).

#### 3.4.14. 2‴-Methoxy *cis*-banglene (**27**)

^1^H NMR (500 MHz, CDCl_3_): δ_H_ 1.64 (2H, m), 2.18 (1H, m), 2.25 (1H, m), 2.75 (1H, m), 3.52 (1H, brs), 3.77 (3H, s), 3.77 (3H, s), 3.78 (3H, s), 3.85 (3H, s), 3.87 (3H, s), 5.52 (1H, dd, *J* = 16.0, 9.2), 5.80 (1H, m, *J* = 10.0 Hz), 5.98 (1H, m, *J* = 10.0 Hz), 6.47 (1H, s), 6.61 (1H, d, *J* = 16.0), 6.72 (1H, d, *J* = 1.7), 6.72 (1H, s), 6.76 (1H, dd, *J* = 8.2, 1.7), 6.80 (1H, d, *J* = 8.2); ^13^C NMR (125 MHz, CDCl_3_): δ_C_ 24.2, 24.9, 42.9, 45.8, 55.7, 55.8, 56.1, 56.3, 56.7, 97.7, 109.4, 110.2, 113.6, 118.8, 122.0, 122.6, 128.0, 129.1, 132.9, 133.9, 143.3, 147.4, 148.0, 148.8, 150.7; EI-MS: *m*/*z* 410 (9), 221 (15), 220 (100), 205 (10), 190 (15), 189 (81), 174 (13), 159 (14).

#### 3.4.15. 2‴-Methoxy *trans*-banglene (**28**)

^1^H NMR (500 MHz, CDCl_3_): δ_H_ 1.68 (1H, m), 1.94 (1H, m), 2.21 (2H, m), 2.39 (1H, m), 3.20 (1H, m, *J* = 8.6 Hz), 3.74 (3H, s), 3.83 (3H, s), 3.85 (3H, s), 3.85 (3H, s), 3.87 (3H, s), 5.68 (1H, m, *J* = 10.0 Hz), 5.90 (1H, m, *J* = 10.0 Hz), 6.02 (1H, dd, *J* = 15.9, 7.6), 6.44 (1H, d, *J* = 15.9), 6.46 (1H, s), 6.72 (1H, d, *J* = 1.9) 6.74 (1H, dd, *J* = 8.2, 1.9), 6.78 (1H, d, *J* = 8.2), 6.88 (1H, s); ^13^C NMR (125 MHz, CDCl_3_): δ_C_ 24.6, 27.9, 45.6, 48.1, 55.8, 55.8, 56.1, 56.5, 56.8, 98.1, 109.6, 110.7, 111.6, 118.8, 120.4, 123.2, 127.6, 130.3, 132.5, 137.7, 143.3, 147.2, 148.5, 148.8, 150.9; EI-MS: *m*/*z* 410 (10), 221 (15), 220 (100), 205 (10), 190 (14), 189 (74), 174 (11), 159 (12).

#### 3.4.16. 2′, 2‴-Dimethoxy *cis*-banglene (**29**)

^1^H NMR (500 MHz, CDCl_3_): δ_H_ 1.75 (2H, m), 2.16 (1H, m), 2.27 (1H, m), 2.80 (1H, m), 3.68 (3H, s), 3.73 (3H, s), 3.79 (3H, s), 3.80 (3H, s), 3.85 (3H, s) 3.86 (3H, s), 4.11 (1H, brs), 5.70 (1H, m, *J* = 10.0 Hz), 5.78 (1H, dd, *J* = 16.1, 8.6), 5.97 (1H, m *J* = 10.0 Hz), 6.44 (1H, s), 6.46 (1H, s), 6.46 (1H, d, *J* = 16.1), 6.71 (1H, s), 6.76 (1H, s); ^13^C NMR (125 MHz, CDCl_3_): δ_C_ 24.2, 25.3, 37.1, 41.5, 56.0, 56.1, 56.3, 56.5, 56.7, 56.8, 96.9, 98.0, 109.5, 114.8, 119.4, 122.3, 122.5, 128.1, 129.6, 131.9, 142.3, 143.2, 147.7, 148.5, 150.7, 151.6; EI-MS: *m*/*z* 440 (7), 221 (15), 220 (100), 205 (10), 190 (11), 189 (77), 174 (12).

#### 3.4.17. 2′, 2‴-Dimethoxy *trans*-banglene (**30**)

^1^H NMR (500 MHz, CDCl_3_): δ_H_ 1.70 (1H, m), 1.90 (1H, m), 2.20 (2H, m), 2.40 (1H, m), 3.73 (3H, s), 3.74 (3H, s), 3.74 (1H, overlapped), 3.84 (3H, s), 3.85 (3H, s), 3.86 (3H, s), 3.87 (3H, s), 5.59 (1H, m, *J* = 10.0 Hz), 5.88 (1H, m, *J* = 10.0 Hz), 6.04 (1H, dd, *J* = 15.9, 8.0), 6.43 (1H, d, *J* = 15.9), 6.45 (1H, s), 6.45 (1H, s), 6.74 (1H, s), 6.92 (1H, s); ^13^C NMR (125 MHz, CDCl_3_): δ_C_ 24.4, 28.1, 39.8, 45.3, 56.1, 56.1, 56.5, 56.6, 56.6, 56.9, 97.5, 98.1, 109.4, 112.6, 119.1, 122.4, 125.0, 127.5, 130.5, 132.9, 142.9, 143.3, 147.5, 148.6, 150.7, 151.4; EI-MS: *m*/*z* 440 (6), 221 (15), 220 (100), 205 (11), 190 (12), 189 (87), 174 (15), 145 (11).

### 3.5. GC–MS Analysis

The pulverized samples were extracted with 1 mL of ethyl acetate per 10 mg sample for 24 h at room temperature. After extraction, the samples were filtered through a 0.45 μm Millipore filter unit (Advantec, Tokyo, Japan) and subjected to GC–MS by injecting 1 μL of sample in the splitless mode. The injector temperature was set at 270 °C, and the carrier gas (helium) was set at a constant flow rate of 1 mL/min. Metabolites were separated using a DB-5MS capillary column (30 m × 0.25 mm i.d., film thickness 0.25 μm, Agilent Technologies, Santa Clara, CA, USA). The GC oven temperature was initially set at 50 °C and held for 3 min, increased to 300 °C at a rate of 10 °C/min, and then maintained at 300 °C for 12 min. Mass spectrometry was performed in the EI mode with an electron energy of 70 eV. The temperature of the ion source and interface was set at 270 °C.

Tentative annotations were performed via library search using the Wiley 9 database. Identifications and annotations were performed according to the confidence levels of metabolite identification defined by the chemical analysis working group of MSI. Briefly, identifications with levels 0 and 1 were isolation and standard, respectively. The structures of isolated compounds were established based on NMR spectroscopic data [41,46,47,48,51,52,53,54,55]. Annotations with level 2 were RI value and EI mass spectral data matched with those in the literature [40].

## 4. Conclusions

In this study, the characterization of the volatile constituents in ethyl acetate extracts prepared from the rhizomes of plai was performed. In the GC–MS TIC chromatograms, thirty major peaks were detected, and their corresponding compounds were annotated or identified. Eventually, twenty-one compounds, including fifteen phenylbutenoids and one phenylbutanoid, were identified by means of isolation procedures or using standard compounds, and nine compounds were annotated on the basis of RI value and EI mass spectral data. Most of the identifications were inconsistent with tentative annotations obtained via library search, indicating the presence of incorrect peak assignments in previous studies. To avoid further misannotations and to contribute to studies on dereplication, the RI value, EI mass spectral data, and NMR data of the isolated compounds are reported.

## Figures and Tables

**Table 1:** Annotation or identification of volatile constituents in the ethyl acetate extracts of plai samples.

Peak	RI	Compounds Annotated via Library Search	Similarity	Compounds Annotated or Identified via This Study	MSI ^1^
**1**	931	α-Thujene (**1**)	95	α-Thujene (**1**)	2
**2**	939	α-Pinene (**2**)	98	α-Pinene (**2**)	2
**3**	979	Sabinene (**3**)	96	Sabinene (**3**)	1
**4**	982	β-Pinene (**4**)	98	β-Pinene (**4**)	2
**5**	991	Myrcene (**5**)	97	Myrcene (**5**)	2
**6**	1019	α-Terpinene (**6**)	96	α-Terpinene (**6**)	2
**7**	1028	*p*-Cymene (**7**)	97	*p*-Cymene (**7**)	2
**8**	1063	γ-Terpinene (**8**)	98	γ-Terpinene (**8**)	2
**9**	1075	4-Thujanol (**9** or **10**)	97	*trans*-4-Thujanol (**9**)	1
**10**	1103	4-Thujanol (**9** or **10**)	96	*cis*-4-Thujanol (**10**)	2
**11**	1188	Terpinen-4-ol (**11**)	96	Terpinen-4-ol (**11**)	1
**12**	1483	3,4-Dimethoxybenzaldehyde (**12**)	93	3,4-Dimethoxybenzaldehyde (**12**)	0
**13**	1530	β-Sesquiphellandrene (**13**)	93	β-Sesquiphellandrene (**13**)	2
**14**	1592	1,4-Dimethoxy-2-methyl-3-(2-propen-1-yl)benzene (**14a**)	86	(*E*)-1-(3′,4′-Dimethoxyphenyl)but-1-ene (**14**)	0
**15**	1633	1,4-Dimethoxytriquinacene (**15a**)	86	(*E*)-1-(3′,4′-Dimethoxyphenyl)buta-1,3-diene (**15**)	0
**16**	1752	δ-Cuparenol (**16a**)	80	Xanthorrhizol (**16**)	1
**17**	1759	Methyl 3,4-dimethoxycinnamate (**17a**)	72	(*E*)-1-(2′,4′,5′-Trimethoxyphenyl)but-1-ene (**17**)	0
**18**	1782	4,7-Dimethoxy-1-indanone (**18a**)	78	(*E*)-3-(3′,4′-Dimethoxyphenyl)propenal (**18**)	0
**19**	1811	1,4,7-Trimethoxytriquinacene (**19a**)	80	(*E*)-1-(2′,4′,5′-Trimethoxyphenyl)buta-1,3-diene (**19**)	0
**20**	1872	1,2-Dimethoxy-4-(1-methoxy-2-propen-1-yl)benzene (**20a**)	76	(*E*)-4-(3′,4′-Dimethoxyphenyl)but-3-en-1-ol (**20**)	0
**21**	1914	2-[(3,4-Dimethoxyphenyl)amino]ethanol (**21a**)	73	Cassumunol H (**21**)	0
**22**	1988	1,4-Dimethoxytriquinacene (**15a**)	76	(*E*)-4-(3′,4′-Dimethoxyphenyl)but-3-en-1-yl acetate (**22**)	0
**23**	3007	(*E*)-1-(3′,4′-Dimethoxyphenyl)buta-1,3-diene (**15**)	76	*cis*-Banglene (**23**)	0
**24**	3048	(*E*)-1-(3′,4′-Dimethoxyphenyl)buta-1,3-diene (**15**)	76	*trans*-Banglene (**24**)	0
**25**	3074	3,6-Dihydro-8,9-dimethoxy-1*H*-2-benzoxocin (**25a**)	73	2′-Methoxy *cis*-banglene (**25**)	0
**26**	3120	3,6-Dihydro-8,9-dimethoxy-1*H*-2-benzoxocin (**25a**)	72	2′-Methoxy *trans*-banglene (**26**)	0
**27**	3134	3,6-Dihydro-8,9-dimethoxy-1*H*-2-benzoxocin (**25a**)	72	2‴-Methoxy *cis*-banglene (**27**)	0
**28**	3172	3,6-Dihydro-8,9-dimethoxy-1*H*-2-benzoxocin (**25a**)	74	2‴-Methoxy *trans*-banglene (**28**)	0
**29**	3195	1-[[(1*E*,3*E*)-4-Methoxy-2-methyl-1,3-butadien-1-yl]thio]-2-methylbenzene (**29a**)	75	2′, 2‴-Dimethoxy *cis*-banglene (**29**)	0
**30**	3239	4-(3,4-Dimethoxyphenyl)-3,4-dihydro-2*H*-pyran (**30a**)	73	2′, 2‴-Dimethoxy *trans*-banglene (**30**)	0

^1^ Confidence levels of identification according to the Metabolomics Standards Initiative (MSI) (0: Isolation; 1: Standard; 2: RI value and EI mass spectral data matched with those in the literature).

## Data Availability

The data presented in this study are available on request from the corresponding author.

## Supplementary Materials

The following supporting information can be downloaded at: https://www.mdpi.com/article/10.3390/molecules29061216/s1, Table S1: Relative contents (%) of major 30 compounds in ethyl acetate extracts derived from the rhizomes of plai purchased in Thailand and Indonesia; Figure S1: EI-MS spectra of isolated compounds.
