# Supplementary material for: Characterization of the Volatile Constituents of Plai (Zingiber purpureum) by Gas Chromatography–Mass Spectrometry
Source: Molecules. 2024 Mar 8;29(6):1216. doi: 10.3390/molecules29061216 (PMC10975921; doi:10.3390/molecules29061216)
Supplement: Supplementary file 1 [file molecules-29-01216-s001.zip › molecules-2883310-supplementary.pdf]

Supporting Information

Characterization of the Volatile Constituents of Plai (*Zingiber purpureum*) by Gas Chromatography–Mass Spectrometry

Yuto Nishidono [1,2,*], Azis Saifudin [3] and Ken Tanaka [1]

1 College of Pharmaceutical Sciences, Ritsumeikan University, Kusatsu 525-8577, Shiga, Japan; ktanaka@fc.ritsumei.ac.jp

2 Research Organization of Science and Technology, Ritsumeikan University, Kusatsu 525-8577, Shiga, Japan

3 Faculty of Pharmacy, Universitas Muhammadiyah Surakarta, Sukoharjo 57102, Jawa Tengah, Indonesia; azis.saifudin@ums.ac.id

* Correspondence: nisidono@fc.ritsumei.ac.jp; Tel.: +81-77-561-5179

List of contents

Table S1. Relative contents (%) of major 30 compounds in ethyl acetate extracts derived from the rhizomes of plai purchased in Thailand and Indonesia. 3

Figure S1. EI-MS spectra of isolated compounds. 4

Table S1. Relative contents (%) of major 30 compounds in ethyl acetate extracts derived from the rhizomes of plai purchased in Thailand and Indonesia.

Peak	Compounds Annotated or Identified via This Study	Relative contents (%)	
		Thailand	Indonesia
**1**	α-Thujene (**1**)	1.1	
**2**	α-Pinene (**2**)	1.0	
**3**	Sabinene (**3**)	10.9	
**4**	β-Pinene (**4**)	1.9	
**5**	Myrcene (**5**)	0.9	
**6**	α-Terpinene (**6**)	1.4	
**7**	*p*-Cymene (**7**)	3.2	
**8**	γ-Terpinene (**8**)	2.1	
**9**	*trans*-4-Thujanol (**9**)	0.8	
**10**	*cis*-4-Thujanol (**10**)	0.8	
**11**	Terpinen-4-ol (**11**)	8.3	
**12**	3,4-Dimethoxybenzaldehyde (**12**)		7.8
**13**	β-Sesquiphellandrene (**13**)	1.9	
**14**	(*E*)-1-(3′,4′-Dimethoxyphenyl)but-1-ene (**14**)	1.3	
**15**	(*E*)-1-(3′,4′-Dimethoxyphenyl)buta-1,3-diene (**15**)	5.9	
**16**	Xanthorrhizol (**16**)		4.5
**17**	(*E*)-1-(2′,4′,5′-Trimethoxyphenyl)but-1-ene (**17**)	1.0	
**18**	(*E*)-3-(3′,4′-Dimethoxyphenyl)propenal (**18**)		6.2
**19**	(*E*)-1-(2′,4′,5′-Trimethoxyphenyl)buta-1,3-diene (**19**)	2.9	
**20**	(*E*)-4-(3′,4′-Dimethoxyphenyl)but-3-en-1-ol (**20**)	9.4	21.5
**21**	Cassumunol H (**21**)		8.5
**22**	(*E*)-4-(3′,4′-Dimethoxyphenyl)but-3-en-1-yl acetate (**22**)	4.2	12.5
**23**	*cis*-Banglene (**23**)	7.6	4.2
**24**	*trans*-Banglene (**24**)	9.4	24.4
**25**	2′-Methoxy *cis*-banglene (**25**)	3.1	
**26**	2′-Methoxy *trans*-banglene (**26**)	3.0	
**27**	2‴-Methoxy *cis*-banglene (**27**)	3.0	
**28**	2‴-Methoxy *trans*-banglene (**28**)	4.5	
**29**	2′, 2‴-Dimethoxy *cis*-banglene (**29**)	3.7	
**30**	2′, 2‴-Dimethoxy *trans*-banglene (**30**)	1.9	

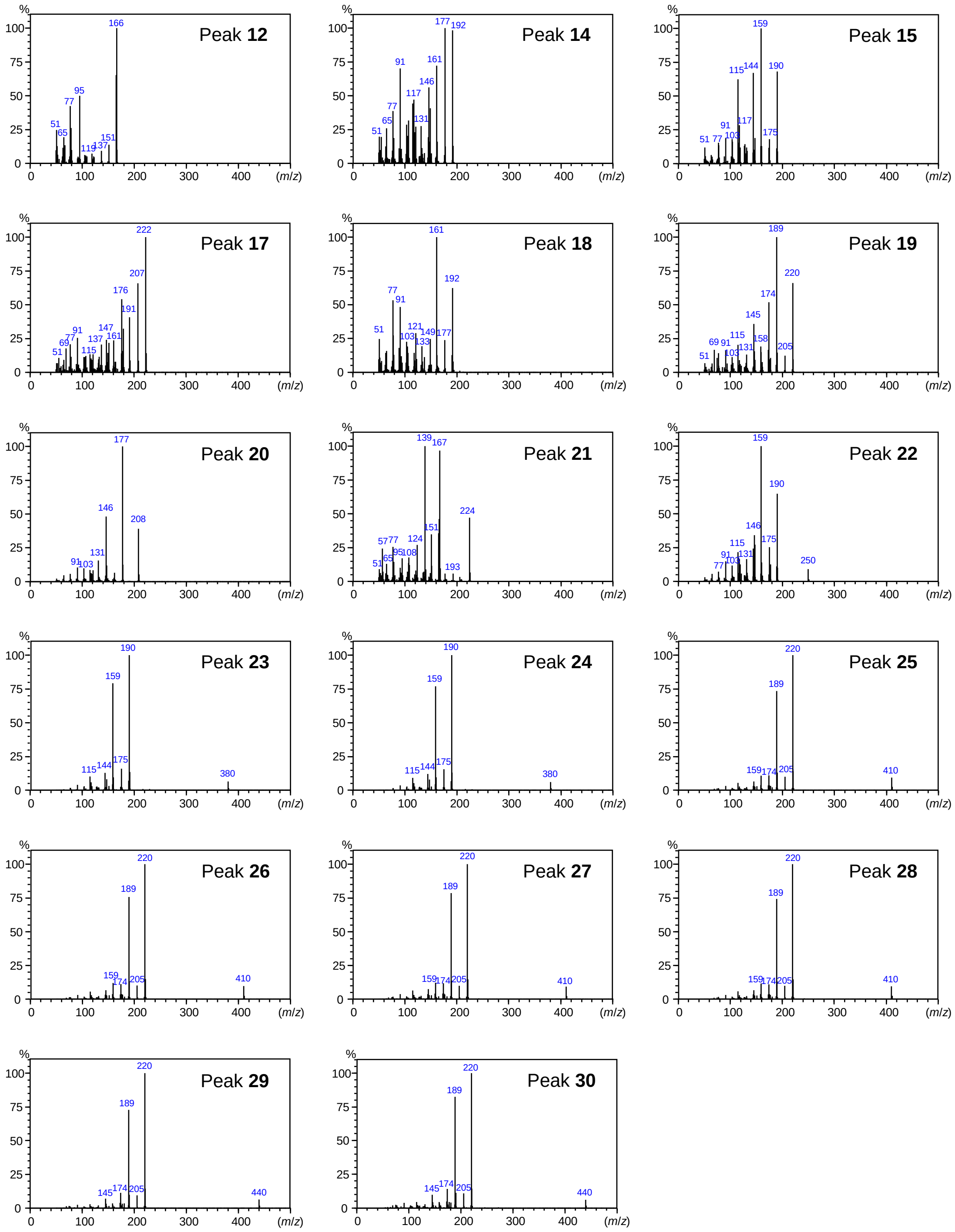

Figure S1. EI-MS spectra of isolated compounds.
